# Fabrication and Characteristics of Heavily Fe-Doped LiNbO_3_/Si Heterojunction

**DOI:** 10.3390/ma12172659

**Published:** 2019-08-21

**Authors:** Wencan Li, Jiao Cui, Dahuai Zheng, Weiwei Wang, Shuolin Wang, Shaoqing Song, Hongde Liu, Yongfa Kong, Jingjun Xu

**Affiliations:** 1 The MOE Key Laboratory of Weak-Light Nonlinear Photonics and TEDA Institute of Applied Physics, Nankai University, Tianjin 300457, China; 2 School of Physics, Nankai University, Tianjin 300071, China

**Keywords:** heavily Fe-doped, conductivity, pulsed laser deposition, heterojunction

## Abstract

A series of heavily Fe-doped LiNbO_3_ (LN:Fe) crystals were grown via the Czochralski method. The dark- and photo-conductivity of the 5.0 wt.% Fe-doped LiNbO_3_ crystal reached 3.30 × 10^−8^ Ω^−1^ cm^−1^ and 1.46 × 10^−7^ Ω^−1^ cm^−1^ at 473 nm, which are about 7 and 5 orders of magnitude higher than that of congruent LiNbO_3_, respectively. Then, a p-n heterojunction was fabricated by depositing the heavily Fe-doped LiNbO_3_ on a p-type Si substrate using the pulsed laser deposition. The current–voltage curve of the LN:Fe/Si heterojunction presents a well-defined behavior with a turn-on voltage of 2.9 V. This LN:Fe/Si heterojunction gives an excellent prototype device for integrated optics and electro-photonics.

## 1. Introduction

The concept of integrated optics has attracted wide interest all over the world, since Yariv suggested integrating photonic devices and electronic devices on the same substrate [1,2] in 1971. Integrating photonic and electronic active and passive components on silicon, now the commonly used substrate in integrated electronics, would make information or energy conversion, transmission, and reception much more efficient. A promising candidate material for such integration with Si substrate is lithium niobate (LiNbO_3_, LN), a well-known nonlinear optical crystal.

LN has many important properties such as electro-optic, acoustic-optic, thermoelectric, piezoelectric, and photorefractive effects. As a strong contender for "optical silicon", LN is applied for optical waveguides, electro-optical modulation, holographic storage, optical parametric oscillators, etc. [3,4,5,6,7,8]. However, LN is generally considered to be an insulator, and acts as a passive component in the above applications [9]. If LN devices can be integrated on silicon substrate to form an active component, then many excellent properties of LN can be applied to semiconductor integrated devices, which will embrace the benefits of both LN and semiconductor materials. The basic unit for an active component is a p-n junction. The low conductivity and absorption of LN were the main challenges posed to manufacturing an active component based on the p-n junction. In order to fabricate a device using LN, it is essential to develop a process that improves the conductivity of LN. The conductivity of LN can be greatly enhanced by introducing Fe into the crystal. However, when the concentration of Fe was below 0.1 wt.%, the conductivity was still very low when compared with semiconductors [10]. Then, it limited the future prospects for ferroelectric integrated devices.

In this work, the photo- and dark-conductivity in LN:Fe were increased several orders of magnitude further than that of the congruent LN (CLN) by heavily doping Fe [11,12]. A heterojunction of LN:Fe deposited on the p-type Si substrate was fabricated with good rectifying properties. This heterojunction provides a promising prospect for the multi-functional applications of passive and active integration. 

## 2. Experimental Details

### 2.1. Samples Preparation

A series of LN crystals doped with 1.0, 3.0 and 5.0 wt% Fe_2_O_3_ (labeled as LN:Fe_1_, LN:Fe_3_ and LN:Fe_5_, respectively) were grown using the Czochralski method. These grown crystals were polarized, cut to 5.0 × 6.0 × 4.5 mm^3^ (X × Y × Z) pieces, and optically polished. In addition, the dopant concentrations given were nominal values, and we cut all the samples from the top of the crystal boule. This ensured as much consistency as possible between the actual and nominal composition of the crystal. In the conductivity measurement, a series circuit was applied on the XY-planes, pasting silver glue as electrodes. The samples were placed in an electrostatic shield to get the dark-conductivity. LN:Fe thin film was deposited on a p-type Si (100) wafer by the pulsed laser deposition (PLD) to obtain a p-n junction. Before deposition, the single side polished Si substrate was ultrasonically cleaned for 10 min in alcohol, acetone, and deionized water, respectively. Then the target of *c*-cut LN:Fe_5_ single crystal and the Si substrate were placed together in a vacuum chamber with a distance of 6.0 cm between them. The chamber was pumped to 10^−5^ Pa and the substrate was heated to 700 °C to keep for 10 min in order to remove the impurities on the surface. The deposition processing lasted 2 h, while the substrate’s temperature of 600 °C and the ambient gas of 30 Pa oxygen were kept. KrF excimer laser operating at 248 nm and 25 ns duration time was used as a light source. The laser with energy density of 1.5 J/cm^2^ was focused at an angle of 45° on the surface of the rotating crystal target. The pulse frequency of the laser was 3 Hz. After deposition, annealing in situ was presented in 10^5^ Pa oxygen presure for 30 min.

### 2.2. Measurements

The absorption of the LN:Fe crystals was measured by a U-4100 Spectrophotometer (Hitachi Science and Technology, Tokyo, Japan). X-ray diffraction (XRD) patterns were measured using a Bruker D8 Advances X-ray Diffractometer (Karlsruhe, Germany). The current-voltage (I-U) curves were measured by a Source Meter (KEITHLEY 6517A, Cleveland, OH, USA). To measure the photoconductivity, a laser with a wavelength of 473 nm and intensity of 1.8 W/cm^2^ passed through the polished XZ-block in a dark room.

## 3. Results and Discussion

### 3.1. Absorption Spectrum and Conductivity of LN:Fe Crystals

The absorption spectra of LN:Fe crystals were measured by a U-4100 Spectrophotometer at room temperature (25 °C). During the measurement, the light is incident vertically onto a sample (0.5 mm optical polished y-cut plate), and we get the transmittance spectrum. Based on the Mclean equation [13], the absorption coefficient (α), transmittance (T), and reflectance (R) satisfy the following relationships:

(1) $$\alpha \left(\lambda \right)=\frac{-1}{d}\ln\left(-b+\sqrt{b^{2}+\frac{1}{R^{2}}}\right)$$

(2) $$R=\frac{{\left(n-1\right)}^{2}}{{\left(n+1\right)}^{2}}$$

(3) $$b=\frac{{\left(1-R\right)}^{2}}{2TR^{2}}$$

*n* is the refractive index of incident light in the crystal, which can be calculated according to the Semeller equation [14]. We can then get the absorption coefficient from the transmittance spectrum. 

**Figure 1:**
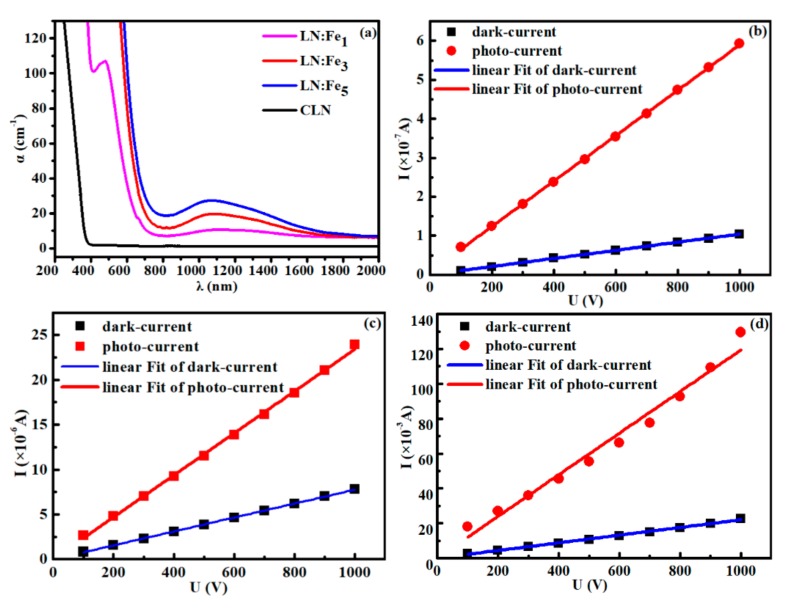
(**a**) The absorption spectra of y-cut LN:Fe and CLN, (**b**–**d**). The current versus voltage (I-U) curves of (**b**) LN:Fe_1_, (**c**) LN:Fe_3_, and (**d**) LN:Fe_5_ blocks, the dark-current in a dark room, and the photo-current with the optical intensity at *λ* = 473 nm were measured, respectively.

As shown in Figure 1a, there is an absorption peak at 477 nm in the spectrum of LN:Fe_1_, while there is an even stronger absorption around the 477 nm in the LN:Fe_3_ and LN:Fe_5_. Compared with LN:Fe_1_, the wide infrared peak of LN:Fe_3_ and LN:Fe_5_ at around 1100 nm is much stronger, which corresponds to the absorption band of free polarons ${\mathrm{Nb}}_{\mathrm{Li}}^{4+}$ [15,16]. Meanwhile, Figure 1a shows that the absorption edges of LN:Fe are redshift with increased doping concentration (from 1.0 wt.% to 5.0 wt.%). In fact, visible and ultraviolet light are almost absorbed in the spectra of LN:Fe_3_ and LN:Fe_5_, and the absorption of infrared light enhances remarkably in LN:Fe_5_.

The results indicate that the absorption region of LN has been enhanced from ultraviolet to infrared band by Fe doping, which makes the excitation of the p-n junction become more selective. In other respects, the heavily Fe-doped LN may be a promising solar energy material, used to improve photoelectric absorption and conversion efficiency due to its excellent optical absorption properties.

The current versus voltage (I-U) curves of LN:Fe are shown in Figure 1b–d. We can see that the photo-current of the LN:Fe is much larger than its dark-current at the same voltage. The dark-conductivity (***σ***_d_) and photo-conductivity (***σ***_ph_) are shown in Table 1. As shown in Figure 1d, especially for LN:Fe_5_, the dark- and photo- conductivity reach 3.30 × 10^−8^ Ω^−1^ cm^−1^ and 1.46 × 10^−7^ Ω^−1^ cm^−1^ respectively, which are about 7 and 5 orders of magnitude higher than that of CLN (5.0 × 10^−15^ Ω^−1^ cm^−1^ for dark-conductivity [11] and 10^−12^ Ω^−1^ cm^−1^ for photo-conductivity [12]).

Based on the above results, the LN:Fe_5_ crystal shows the nice absorption and enhanced conductivity properties, which may be contributed to the concentration of Fe^3+^ and Fe^2+^ and their ratio in LN. However, as the Fe doping concentration increases, it becomes difficult to grow high quality doped LN crystals. It is worth increasing the Fe doping concentration, which we can try to realize through improving the crystal growth process or preparing the target using the solid-state sintering of powder method. In addition, the results attract us to construct a heterojunction with the n-type LN layer and the p-type Si layer, which provides the possibility of integrated optical devices based on LN.

### 3.2. LN:Fe_5_/Si p-n Junction and Its Rectification Characteristics

**Figure 2:**
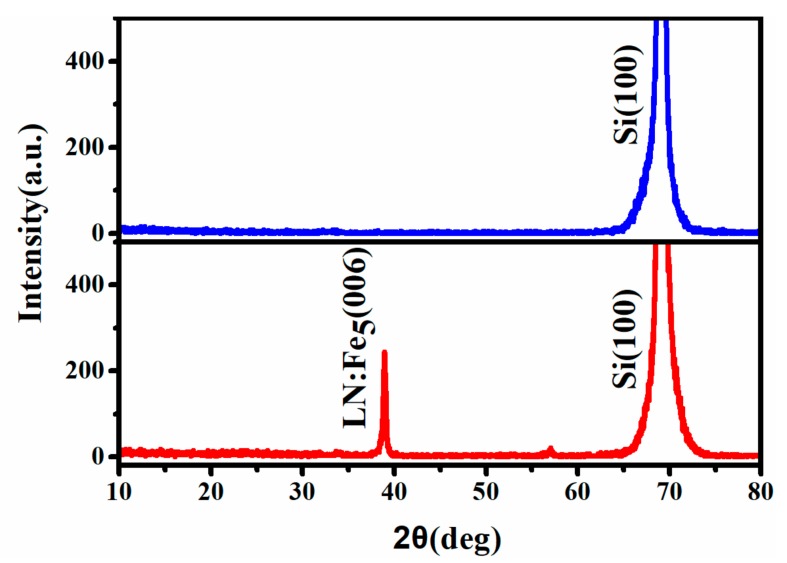
X-ray diffraction (XRD) patterns for LN:Fe_5_/Si p-n junction, the diffraction peak (above) at 2θ = 69.1°corresponds to the Si(100) substrate and the peaks labeled LN:Fe_5_(006) and Si(100) (below) correspond to LN:Fe_5_ thin film at 2θ = 39.1°and Si substrate 2θ = 69.1°.

A p-n junction of LN:Fe_5_/Si was manufactured by PLD. The film thickness controlled by deposition time is about 2.0 µm, which can be measured by a step profiler. The XRD patterns of the substrate (above) and the p-n junction (below) are shown in Figure 2. Si(100) peak of the substrate is found at 69.1°. Except for the peak of the substrate, a stronger peak of LN:Fe_5_(006) at 39.1° was simultaneously measured. The full width half maximum (FWHM) of the (006) oriention of LN:Fe_5_ is about 0.37°, which is less than the FWHM value (1.4°) of the undoped films of LN single crystal [17]. The results indicate *c*-axis oriented LN film with very few flaws was successfully deposited on the Si substrate.

**Figure 3:**
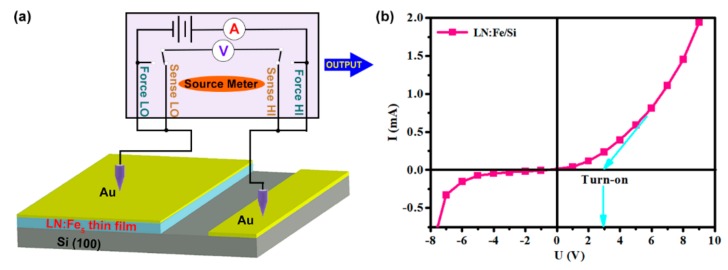
(**a**) Schematic diagram of the experimental setup for p-n junction LN:Fe_5_/Si. (**b**) The current-voltage curve of the LN:Fe_5_/Si junction with a turn-on voltage of 2.9 V for the forward voltage and a breakdown voltage of about −6 V.

Figure 3a is the schematic diagram of the experimental setup of LN:Fe_5_/Si p-n junction for measuring current-voltage (I-U), and its rectifying property is shown in Figure 3b. As the film is very thin, the relaxation time of the p-n junction is less than 10 seconds and the LN:Fe_5_/Si p-n junction is stable. Obviously, good rectifying property with a turn-on voltage of 2.9 V for the forward voltage was measured. A breakdown voltage of −6 V was also shown in Figure 3b.

Based on the above results, we can find that the LN:Fe_5_/Si presents obvious rectifying characteristics with a turn-on voltage of 2.9 V and a breakdown voltage of −6 V. For a typical SOI-type p-n junction, which exhibits a turn-on voltage of 2.2 V and a breakdown voltage of 550–770 V [18], many factors need to be considered in evaluating its performance. In any case, for the LN:Fe_5_/Si, the turn-on voltage and the breakdown voltage are less desirable. Further research is ongoing to optimize the heterojunction performance.

### 3.3. Energy Band Diagram of the LN:Fe_5_/Si p-n Junction 

**Figure 4:**
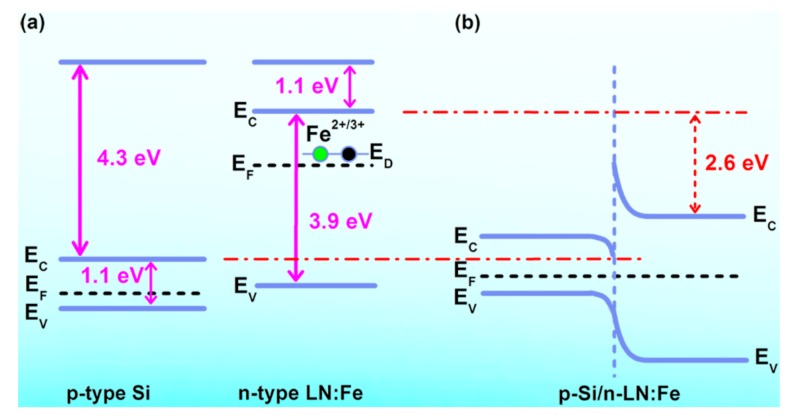
(**a**) Energy band diagram of isolated p-type Si and n-type LN:Fe_5_. (**b**) Energy band diagram of ideal LN:Fe_5_/Si heterojunction at thermal equilibrium.

To understand the rectifying property, the energy band diagram of the p-n junction was studied. Figure 4a shows the energy band diagram of isolated p-type Si and n-type LN:Fe_5_. The work function of heavily doped p-type Si was reported as *W_Si_ =* 5.00 eV [19] and the band gap of Si is approximately 1.11 eV [20]. CLN film deposited by PLD is usually Li-deficient and the band gap and electron affinity (*χ*) of LN were still regarded as 3.90 eV and 1.10 eV [21,22,23]. That is to say, the impurity defects in LN do not change its intrinsic band gaps and electron affinity energy significantly, but they introduce defect level. So we consider that the band gaps and electron affinity energy of LN:Fe_5_ are approximately 3.90 eV and 1.10 eV, and the gap (*E_n_*) between condution band (*E_C_*) and fermi level (*E_F_*) of the LN:Fe_5_ is as follows:

(*E_n_*)*_LN:Fe5_* = *E_C_* − *E_F_* = 1.30 eV (4)

The work function of LN:Fe_5_ is approximately calculated according to Equation (5):

*W_LN:Fe5_* = χ + (*E_n_*)*_LN:Fe5_* = 2.40 eV (5)

As shown in Figure 4b, when two kinds of materials are combined, the energy band bending occurrs. The value of the bending energy is determined on the work function according to Equation (6):

*ΔE*= *W_Si_* − *W_LN:Fe5_* = 2.60 eV (6)

Since the carriers in Si are much more than that in LN, band bending falls mainly in LN and forms the potential barrier, which is close to the value of turn-on voltage 2.9 V. In other words, an energy of 2.60 eV is necessary to get a forward current. 

## 4. Conclusions

In summary, up to 5.0 wt.% Fe_2_O_3_ doped LiNbO_3_ crystals were grown. The conductivity of LN:Fe was greatly increased by 7 (dark-conductivity) and 5 (photo-conductivity) orders of magnitude compared with CLN. A p-n junction of LN:Fe_5_/Si was fabricated via the PLD method. The LN:Fe_5_/Si presents the rectifying characteristic with a turn-on voltage of 2.9 V for the forward voltage and a breakdown voltage of −6 V for the backward voltage. The energy band diagram indicates that an energy of 2.60 eV is necessary to get a forward current. The LN:Fe_5_/Si heterojunction gives an excellent prototype device for integrated optics and electro-photonics. 

## Figures and Tables

**Table 1:** The dark- and photo-conductivity of LN doped with 1.0, 3.0, and 5.0 wt.% Fe_2_O_3_. A 473 nm laser with light intensity of 1.8 W/cm^2^ was applied to the measurement of photo-conductivity.

Crystal	*σ*_d_ (Ω^−1^ cm^−1^)	*σ*_ph_ (Ω^−1^ cm^−1^)
**LN:Fe_1_**	1.56 × 10^−10^	7.70 × 10^−10^
**LN:Fe_3_**	1.16 × 10^−8^	2.35 × 10^−8^
**LN:Fe_5_**	3.30 × 10^−8^	1.46 × 10^−7^
**CLN**	5.0 × 10^−15^ [11]	10^−12^ [12]
